# Immunogenic Targets for Specific Immunotherapy in Multiple Myeloma

**DOI:** 10.1155/2012/820394

**Published:** 2012-05-07

**Authors:** Lu Zhang, Marlies Götz, Susanne Hofmann, Jochen Greiner

**Affiliations:** ^1^Department of Internal Medicine III, University of Ulm, 89081 Ulm, Germany; ^2^Department of Oncology, Tongji Hospital, Tongji Medical College, Huazhong University of Science and Technology, 430000 Wuhan, China

## Abstract

Multiple myeloma remains an incurable disease although the prognosis has been improved by novel therapeutics and agents recently. Relapse occurs in the majority of patients and becomes fatal finally. Immunotherapy might be a powerful intervention to maintain a long-lasting control of minimal residual disease or to even eradicate disseminated tumor cells. Several tumor-associated antigens have been identified in patients with multiple myeloma. These antigens are expressed in a tumor-specific or tumor-restricted pattern, are able to elicit immune response, and thus could serve as targets for immunotherapy. This review discusses immunogenic antigens with therapeutic potential for multiple myeloma.

## 1. Introduction

Multiple myeloma (MM) will account for an estimated 20,180 new cancer cases in the United States in 2010, including 11,170 cases in men and 9,010 cases in women, with an estimated 10,650 deaths [1]. The implementation of autologous stem-cell transplantation and novel agents such as thalidomide, lenalidomide, and bortezomib has changed the management of myeloma and extended overall survival [2–4]. The 5-year survival rate reported in the Surveillance Epidemiology and End Results database has increased from 25% in 1975 to 34% in 2003 owing to those newer and more effective treatment options available [5, 6]. However, patients invariably relapse even if complete remission is achieved. Patients in relapse, usually in old ages [7] and having experienced a long term of treatment, have poor tolerability to further intervention, whereas the myeloma cells have acquired resistance to previous therapy and are very likely cross-resistant to similar drugs. Therefore, the final outcome of patients with MM is sad, and MM still remains an incurable disease. Novel therapeutics are still being expected to further improve the outcome of patients with MM.

Antitumor immunotherapy is proved to be well-tolerated and thought to be unlikely cross-resistant with current drugs and thus might be a powerful intervention to maintain a long-lasting control of minimal residual disease or to even eradicate disseminated tumor cells [8, 9]. Some immunotherapies have achieved clinical success and have been approved for clinical use in tumors, employing monoclonal antibodies or immune cells. For example, rituximab, an anti-CD20 antibody, has extended survival of patients with B-cell non-Hodgkin's lymphoma (NHL). Medication of rituximab as single treatment or in combination with chemotherapy has been a standard treatment for NHL. [10, 11]. Sipuleucel-T is an active cellular immunotherapy consisting of autologous PBMCs pulsed in vitro with a tumor-associated antigen. Benefit from Sipuleucel-T was confirmed in patients with castration-resistant prostate cancer by a phase III randomized trial [12] and Sipuleucel-T has become the first cellular therapeutic for solid tumors approved by FDA. However, immunotherapy with unequivocal clinical benefit is not established yet in MM. Several tumor-associated antigens have been identified in patients with MM. Some of them are expressed in a tumor-specific or tumor-restricted pattern and are able to elicit immune response. Thus, they might be the candidate of targets for immunotherapy. This review discusses immunogenic antigens which are present in MM and have therapeutic potential for patients with MM.

## 2. Targets for Specific Immunotherapy in MM

### 2.1. Immunogenic Antigens

A few but growing number of immunogenic antigens have been discovered in MM, including idiotypes on MM immunoglobulin, MUC1, WT1, a subgroup of cancer-testis antigens (CTAs), receptor for hyaluronic acid-mediated motility (RHAMM), Dickkopf1 (DKK1) and HM1.24. Idiotype refers to the unique immunological properties of any individual immunoglobulin. Normally each B-cell clone synthesizes one certain type of immunoglobulin which is unique to the B-cell. As MM is a clonal B-cell malignancy, idiotype has been considered a tumor-specific and even individual-specific antigen [53]. The other immunogenic antigens are shared with other solid tumors or hematologic malignancies. These antigens are able to elicit humoral and cellular immune reactions in patients with MM (discussed below), and most of them are linked to cell cycle or proliferation [13, 17, 22, 26, 35, 39, 45] (Table 1). Therefore, these antigens are considered competent target structures for immunotherapy.

### 2.2. Immunotargets Expressed in MM

The expression of those immunogenic antigens mentioned above is frequent in MM cells and restricted in normal tissue, which is very suitable for immunotargets. Idiotype [13], RHAMM [36], DKK1 [39, 40], and HM1.24 [46] are expressed in almost all MM patients; MUC1 [18], WT1 [23], and MAGE-C1 [27–30] are expressed in the majority of MM patients and ropporin is detected in about 44% of MM patients [33]. In normal tissue their expression is restricted [13, 24, 31, 34, 35, 40, 41, 46], with the exception of MUC1. MUC1 is ubiquitously expressed on the luminal surface of most simple epithelial cells. However, on malignant cells MUC1 is overexpressed and aberrantly glycosylated [19] and thus can be distinguished from MUC1 on normal cells. Notably, HM1.24 had been thought to be preferentially expressed on terminally differentiated B-cells and overexpressed on MM cells, but not or slightly expressed in other normal tissues [46]. However, a recent report questioned the expression pattern of HM1.24 and the rationality of HM1.24 as a target for immunotherapy. Erikson et al. investigated the expression of HM1.24 by tissue microarray and found that it was expressed in a number of normal cell types including hepatocytes, pneumocytes, pancreas and kidney, epithelia, monocytes, and vascular endothelium [45]. This discrepancy in expression profile was ascribed to differences in the sensitivity of immunodetection. Resulting from this, the expression profile of HM1.24 and the safety of HM1.24 targeting immunotherapy need to be reconsidered.

### 2.3. Specific Immune Response against Immunogenic Antigens in MM

As the first identified tumor-associated antigen in MM, idiotype has been proved to be immunogenic by plenty of experiments, and the immunogenicity was further confirmed in clinical trials [13–16]. An HLA-A2-restricted peptide derived from MUC1 was used to pulse dendritic cells (DCs) and generated specific cytotoxic T lymphocytes (CTLs). These specific CTLs exhibited cytotoxicity against a myeloma cell line expressing MUC1. Another peptide derived from hTERT also showed a similar effect in this experiment [21]. Spontaneous formation of antibodies against MUC1 was reported in MM patients, although at a low level [20].

CTA-specific immune responses were also reported in MM patients. Spontaneous antibodies and T lymphocytes directed against NY-ESO-1 were detected in MM patients. After being expanded by autologous antigen presenting cells (APCs) pulsed with NY-ESO-1-derived peptide analog, the specific CTLs were able to lyse primary MM cells [54]. Ropporin is a novel CTA identified in 2007 [34]. Specific antibodies were detected in the serum of all patients who showed ropporin protein staining in immunocytochemistry. Furthermore, ropporin protein was proved to be located on the surface of MM cells, suggesting that ropporin could be exploited as a target for antibody therapy. Moreover, specific CTLs were generated by incubation with autologous DCs loaded with ropporin and showed cytolytic effect against autologous MM cells [33].

MAGE-C1 is the most commonly expressed CTA in MM. Recently several studies investigated the immunogenicity of MAGE-C1 in MM patients. Lendvai et al. first described CD8+ T-cell reactions against MAGE-C1, which were detected in MM patients, and these CD8+ T-cell responses were restricted to patients expressing MAGE-C1 mRNA in their CD138+ myeloma cells. However, the sample size of this study was small, and more extended data from further samples have to be generated [26]. HLA-A2-restricted epitopes have been identified from MAGE-C1, and specific CD8+ T cells were able to recognize MAGE-C1 expressing myeloma cells [31], which may facilitate development of immunotherapy targeting MAGE-C1. CD4+ T-cell reactions against MAGE-C1, which are indispensable for a robust immune effect, were also reported, although not yet in MM patients [55]. In addition, specific antibodies against MAGE-C1 were detected in 50% of MM patients and in nearly all patients with MAGE-C1 expressing myeloma cells, demonstrating a high immunogenicity of epitopes derived from MAGE-C1 [32]. However, in the study of Lendvai et al., simultaneous humoral immune responses were not detected, probably because of the small sample size [26].

Dickkopf1 (DKK1) is a secreted protein that hampers bone formation by inhibiting the Wnt/*β*-catenin pathway and thus contributes to osteolytic bone disease in MM [56]. Qian et al. identified HLA-A2-restricted peptides from DKK1 and proved the immunogenicity by vaccinating HLA-A*0201 transgenic mice. Corresponding specific CTL precursor cells were detected in MM patients, although at a low frequency. Stimulation by autologous DCs loaded with DKK1 peptides generated specific T cells which were able to lyse DKK1-expressing cells including autologous primary MM cells, in an HLA-A2 restricted manner [40]. In another recent report, the efficiency of DKK1-DNA vaccine was examined in a murine MM model. The vaccination elicited a strong and specific CD4+ and CD8+ T-cell response. Moreover, the vaccine was able to protect mice from MM challenge and exhibited therapeutic effect against established MM [44]. Two anti-DKK1 neutralizing antibodies have been tested as therapeutic agents in mice bearing human primary MM. Both antibodies reduced osteolytic bone resorption and increased bone formation, indicating their potential application for the palliative treatment. Furthermore, the two antibodies also inhibited myeloma cell growth *in vivo*, probably through blockage of bone marrow stromal cell/MM cell adhesion and production of IL-6 [42, 43].

HM1.24 antigen (also referred to as CD317, BST2, or tetherin) is a surface molecule involved in controlling virus infection [45]. HM1.24-loaded DCs were able to induce specific CTLs *in vitro* from peripheral blood of healthy volunteers and MM patients. The specific CTLs showed the ability to recognize and lyse myeloma cells [50–52]. Several HLA I-restricted epitopes within HM1.24 have been identified and proved to be of potent immunogenicity [52, 57]. More evidence of humoral immune responses is available by targeting HM1.24. A humanized anti-HM1.24 monoclonal antibody has been developed and has exhibited antimyeloma effect by inducing antibody-dependent cellular cytotoxicity (ADCC) [47]. Injection of this antibody inhibited tumor growth, reduced tumor load, and prolonged survival of myeloma-bearing mice in xenograft mouse model [48]. Defucosylation could further enhance the ADCC against primary myeloma cells [49].

### 2.4. Clinical Trials against Immunotargets

Idiotype, WT1, and RHAMM have been tested as therapeutic targets in published clinical trials. Several studies have investigated the use of idiotype protein or peptide pulsed DCs as vaccine for patients with MM [16]. Immune responses were evoked in some patients, but clinical responses were rare. This unsatisfactory outcome of idiotype vaccination in MM was partially due to the weak immunogenicity of idiotype proteins [58]. Different approaches are under clinical evaluation for enhancing idiotype-targeted immune response [59, 60].

WT1 peptide-based vaccination was performed in a patient with advanced chemotherapy-resistant MM. The frequency of WT1 specific CTLs increased after vaccination. Myeloma cells in BM decreased from 85% to 25%, and the amount of M protein in the urine decreased from 3.6 to 0.6 g/day. A bone scintigram showed improvement of bone lesions, especially of the ribs, so that the clinical response was assessed as minimal (EGBMT criteria) [25].

HLA-A2-restricted epitopes within RHAMM have been identified [61], and the most robust one, which was designated R3, was tested in clinical peptide vaccination trials in patients with acute myeloid leukemia, myelodysplastic syndrome, and MM. Seven patients with MM were recruited and exhibited exciting results. Immune responses were observed in 6/7 (85.7%) patients and three of them showed a positive clinical effect, which was manifested by reduction of free light chain serum levels [37, 38]. The high frequency of immune responses and clinical responses indicates that RHAMM could be a promising target for T-cell therapy against MM.

Furthermore, several clinical trials are ongoing to investigate the vaccination effect of NY-ESO-1 or MAGE-A3 peptide with GM-CSF (NCT00090493), MUC1 peptide with GM-CSF (NCT01232712), or idiotype-KLH and T-cells (NCT01426828) in MM patients (http://www.clinicaltrials.gov/). These clinical trials will yield more evidence of specific immunotherapy for MM using different targets.

## 3. Future Perspectives

Different immunogenic targets expressed in MM have to be evaluated in future clinical trials to detect their clinical relevance. Candidates could be antigens identified from MM, or antigens which have shown therapeutic potential in other malignancies like MUC1 and WT1. CD4+ T-cell activation, which is indispensable for inducing efficient immune effect, was described for idiotype [15], MUC1 [21], and DKK1 [44] in MM. Therefore, the ability to stimulate CD4+ T-cells and the corresponding epitopes of the immunogenic targets need to be further investigated. Adjuvants help breaking immune tolerance in MM patients and enhance immune effects, but the optimal adjuvant has to be established for individual antigens [59]. Bivalent or multivalent vaccines against different antigens might be another strategy to strengthen immune response and prevent immune evasion.

## 4. Summary

There is an urgent need for novel therapeutics to improve the outcome of patients with MM. An increasing number of immunogenic antigens have been discovered in MM and thus open the possibility to develop specific immunotherapy for MM. Some of them have been tested in clinical vaccination trials and generated important results. Immunotherapy targeting these antigens might be a promising approach to reduce or delay relapse, and thus improve outcome of MM.

## Figures and Tables

**Table 1:** Expression profile and immune responses of promising immunotargets in MM.

Antigen	Function	Expression in MM	Expression in normal tissue	Humoral response in MM	CD8+ T-cell response in MM	CD4+ T-cell response in MM	Clinical trials in MM
Idiotype	Essential for B-cells function and survival [13]	Nearly 100% [13]	B-cells [13]	Yes [14]	Yes [13, 15]	Yes [15]	Phase I-II, clinical response was disappointing [16]

MUC1	Multiple functions including surface barrier, signal transduction, and so forth [17]	Fully glycosylated: 73% Differentiation-dependent glycoforms: 59% Cancer-associated glycoforms: 36% [18]	Ubiquitous on the luminal surface of most simple epithelial cells [19]	Yes [20]	Yes [21]	Yes [21]	ND

WT1	Transcription factor [22]	Frequent but at a low level [23]	Placenta [24]	ND	Yes [25]	ND	One patient reported, showing decreased myeloma cells [25],

MAGE-C1	Probably dysregulation of the cell cycle [26]	70–80% [27–30]	Testis, placenta [31]	Yes [32]	Yes [26]	ND	ND

Ropporin	Unknown	44% [33]	Testis [34]	Yes [34]	Yes [33]	ND	ND

RHAMM	Formation of mitotic spindle, signal transduction [35]	100% [36]	Testis, placenta, thymus [24, 35]	ND	Yes [37, 38]	ND	Two phase I/II peptide vaccination trials, including 7 MM patients, with three showing clinical response [37, 38]

DKK1	Inhibitor of osteoblast differentiation [39]	Almost all patients [40]	Placenta, prostate and testis [40, 41]	Yes [42, 43]	Yes [40, 44]	Yes [44]	ND

HM1.24	Antiviral restriction factor [45]	100% [46]	Terminally differentiated B-cells* [46]	Yes [47–49]	Yes [50–52]	ND	ND

*The expression of HM1.24 in normal tissue needs to be further investigated.

ND: No data available.
